# A longitudinal investigation of the factors associated with increased RISk of playing-related musculoskeletal disorders in MUsic students (RISMUS): a study protocol

**DOI:** 10.1186/s12891-019-2440-4

**Published:** 2019-02-08

**Authors:** Cinzia Cruder, Pelagia Koufaki, Marco Barbero, Nigel Gleeson

**Affiliations:** 1 Rehabilitation Research Laboratory 2rLab, Department of Business Economics, Health and Social Care, University of Applied Sciences and Arts of Southern Switzerland, Manno, Switzerland; 2 Department of Research and Development, Conservatory of Southern Switzerland, Lugano, Switzerland; 3 Queen Margaret University, Centre of Health, Activity and Rehabilitation Research, Edinburgh, United Kingdom

**Keywords:** Playing-related musculoskeletal disorders, Longitudinal study, Study protocol, Risk factors, Music students

## Abstract

**Background:**

The achievement and improvement of skills in musical techniques to reach the highest levels of performance may expose music students to a wide range of playing-related musculoskeletal disorders (PRMDs).

In order to establish effective solutions for PRMDs and to develop future preventive measures, it is fundamental to firstly identify the main risk factors that play a significant role in the development of musculoskeletal conditions and symptoms.

The aim of the study is to identify those factors associated with increased risk of PRMDs among music students.

A further goal is to characterise this population and describe the clinical features of PRMDs, as well as to determine the evolving course of PRMDs in music students during their training.

**Methods:**

One hundred and ninety schools have been invited to participate in this study, sixty of which have already confirmed officially their support for the investigation’s recruitment procedures, by means of a subsequent distribution of the link to a web-based questionnaire to their student groups (total potential student numbers available: *n* = 12,000 [based on ~ 200 students per school on average, and 60 volunteering schools]; expected number of students: *n* = 3000 [based on a 25% response rate from the 12,000 students attending the 60 volunteering schools]).

The web-based questionnaire includes questions about any PRMD that students have experienced during their training, and different potential risk factors (i.e. lifestyle and physical activity, practice habits, behaviour toward prevention and health history, level of stress, perfectionism, fatigue and disability).

Overall recurrence or new onsets of PRMDs will be assessed at 6 and 12 months after the first data collection to investigate and record the development of new incidents within a period of a year and to enable characterisation of the nature and the evolving course of PRMDs.

**Discussion:**

To the best of our knowledge, no other longitudinal studies on risk factors for PRMDs among music students have been conducted so far. Therefore, this study can be considered as an opportunity to begin filling the gaps within current research in this field and to generate new knowledge within musical contexts in education and employment.

**Trial registration:**

ClinicalTrials.gov (NCT03622190), registration date 09/08/2018.

**Electronic supplementary material:**

The online version of this article (10.1186/s12891-019-2440-4) contains supplementary material, which is available to authorized users.

## Background

It has been shown that professions with high physical demands and/or frequently repeated movements like tool use (i.e. people primarily engaging in activities with manual handling) are associated with a higher prevalence of musculoskeletal problems [1–3]. Similarly, professional musicians are exposed chronically to large amounts of continuous and repeated physical movements and are vulnerable to developing musculoskeletal conditions and symptoms [4–6] that may affect the manner in which, and the extent to which music can be practised and performed [6, 7]. Indeed, this phenomenon was described by Zaza et al. [8] as playing-related musculoskeletal disorders (PRMDs) and defined as “any pain, weakness, numbness, tingling or other symptoms that interfere with the ability to play your instrument at the level you are accustomed to”. This definition does not include mild transient aches or pains. In fact, according to Zaza et al. [8], musicians use the words mild, just, slightest, normal and little to describe such aches and pains that would not be considered to be related to PRMDs, because they don’t affect the ability to play the musical instrument and are considered as “normal” everyday pain [8].

Current literature suggests that PRMDs do not only occur when entering the professional world but slowly develop over time starting from the early stages of advanced musical training and education. Between 25 and 43% of music students at university level, admitted that they had experienced PRMDs before starting their degree course [9] or had experienced a health problem related to their activity as musicians during the early stages of their education [10]. Brandfonbrener [11] reported a prevalence of playing-related pain to be approximately 85% among first year music students at university level and found that the majority had already experienced PRMDs as pre-college students, or when even younger.

The first year of a degree course in music is particularly demanding [12] and the transition from pre-college- to university-level studies requires intensified practice. This is indispensable primarily because students need to achieve higher instrumental performance capabilities and perform in a more competitive physical and psychosocial environment involving different techniques and performances introduced by new professors and teachers.

Preventive programs and health promotion should be implemented at the beginning of their musical training, with the objective to protect music students from PRMDs during their studies and to prepare them for future professional demands.

In order to find effective solutions to prevent or minimise the development of disorders and consequences for music students, it is fundamental to firstly identify the main risk factors that contribute to the development of PRMDs.

Currently available studies offer very limited appraisal of possible relationships between the musician’s performance demands and the development of disorders due to limitations in the research designs that had been used (i.e. lack of longitudinal observations), low methodological quality (i.e. high measurement bias, inappropriate statistical analysis) and large heterogeneity amongst the assessment approaches and outcomes [13].

The existing literature on risk factors among musicians cannot be considered acceptable for the purpose of establishing definitive links between specific characteristics of musicians and their risks of developing PRMDs. This is because the available studies are predominantly cross-sectional and terms such as prognostic factors or predictors are inappropriately used to indicate associations [13, 14].

Several recent studies and reviews recommended conducting a longitudinal investigation with a combination of biological, psychological and social factors that contribute to the development of PRMDs [13–18].

It is plausible that with more evidence relating to modifiable factors that may increase the risk of adverse outcomes, targeted behaviour-modification and health-promotion might be ultimately designed to counteract the risk of developing PRMDs among music students.

Thus, relevant targeted interventions could be implemented at the initiation of music students’ training, or delivered as intermediate or ongoing interventions during, or in the transition towards, professional musicianship.

### Aims of the study

The aim is to identify those factors most strongly associated with increased risk of PRMDs among music students undertaking professional training.

A further goal is to characterise this population and describe the clinical features of PRMDs, as well as to determine the evolving course of PRMDs in music students during their professional training.

## Methods

### Design

This longitudinal study is to be conducted in order to obtain self-reported data from a large population of music students of different European university schools of music at baseline, and then at 6 months and 12 months follow-up.

The study protocol has been approved by the Research Ethics Panel of the Queen Margaret University of Edinburgh (REP 0177).

### Study centres and participants

One hundred and ninety schools have been invited to participate in this study, sixty of which have already confirmed officially their support for the investigation’s recruitment procedures, by means of a subsequent distribution of the link to a web-based questionnaire to their student groups (total potential student numbers available: *n* = 12,000 [based on ~ 200 students per school on average, and 60 volunteering schools]; expected number of students: *n* = 3000 [based on a 25% response rate from the 12,000 students attending the 60 volunteering schools]).

The recruitment e-mail contains information about the study, a participant information sheet and the link to the web-based questionnaire site. The link directs interested students to an electronic written consent form, which has to be completed and signed before they would be permitted to proceed to the completion of the web-based questionnaire.

Recruitment bias has been minimised because the researchers have no connection with the students until they provide informed consent and complete the web-based questionnaire.

Furthermore, confidentiality is ensured by assigning a unique identification code number to every participant who completes the web-based questionnaire. Personal information provided through informed consent and participants’ data collected through the web-based questionnaire, will be stored separately.

Inclusion criteria include: 1) Pre-college students in years 3 or 4; 2) University-level students in years 1, 2 and 3 of a Bachelor’s degree course; 3) Master of Arts students in years 1, 2, 3; and 4) men and women, aged over 18 years old with a musical instrument commonly used in classical music as main subject.

Exclusion criteria include: 1) composers and conductors; 2) positive history of neurological and/or rheumatic and/or psychological disorders in the last 12 months; 3) surgery of the upper limbs and/or the spine in the last 12 months.

### Procedure

**Fig. 1:**
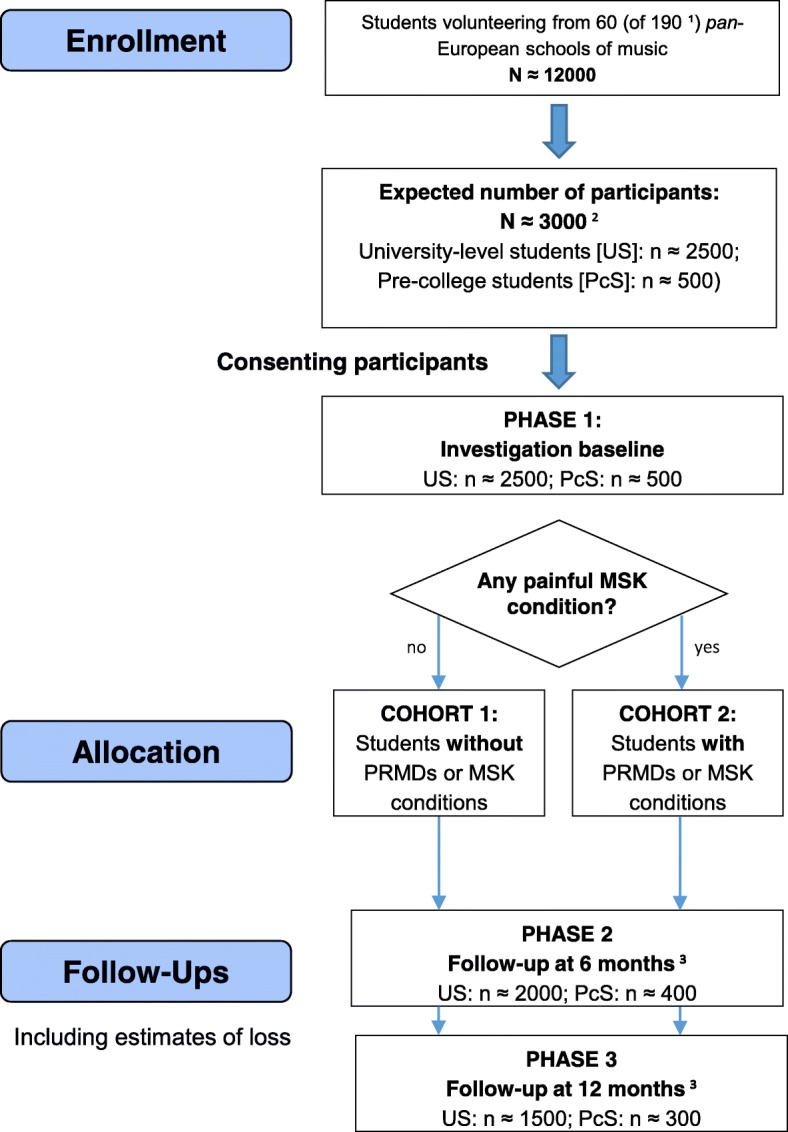
Research planning chart including main features of the protocol study. ^1^ Total of 190 schools within Europe and meeting inclusion criteria; *n* = 60 schools already offering facilitated contact with candidate participants; ^2^ Estimated using a response rate of 20% and based on an invitation to participate to 200 students per school, on average, within 60 schools; ^3^ Estimated using a loss-to-follow-up of 20 and 25% at Phase 2 (6 months) and Phase 3 (12 months), respectively. MSK, musculoskeletal; PRMDs, playing-related musculoskeletal disorders

The study will be conducted through the following three phases (See Fig. 1):

1. Phase 1: baseline cross-sectional description of the study population
2. Phase 2: 6-months follow-up investigation, and
3. Phase 3: 12-months follow-up investigation

After the first phase and for further analysis, the students will be segregated into two separate cohorts:

- Cohort 1: music students (both pre-college and university-level) who have not experienced PRMDs and/or other painful musculoskeletal (MSK) conditions in the last 12 months
- Cohort 2: music students (both pre-college and university-level) who have experienced PRMDs and/or other painful MSK conditions in the last 12 months

Phase 1 will be conducted to describe the prevalence of PRMDs and other painful MSK conditions in the study population. It also aims to compare differences in PRMDs and associated variables between subgroups in Cohort 2.

Subgroups’ analysis will include: women and men, instrument groups, pre-college vs university-level, undergraduate vs postgraduate.

Afterwards, the two cohorts will be followed and invited for reassessment at 6 months (i.e. Phase 2) and 12 months (i.e. Phase 3).

The 6-months and 12-months follow-up for the Cohort 1 will be essential to monitor and record the development of new cases of PRMDs within a period of a year and to enable characterisation of the nature and time-course of developing PRMDs.

Similarly, the follow-ups for the Cohort 2 will be important in order to describe the time-course of any PRMD change/progression within a 12-month period.

It also aims to facilitate optimised sample size for multivariate analysis, to enable subgroups’ analysis and to develop predictive models of risk factors influencing the severity and extent of existing PRMDs.

### The research investigation

**Fig. 2:**
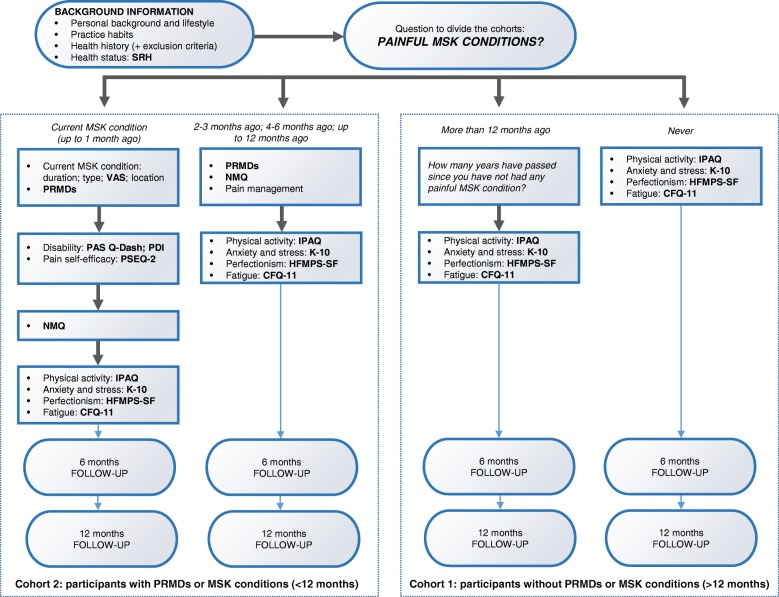
Procedure of the web-based questionnaire of the protocol study. SRH, Self-Rated Health; MSK, musculoskeletal; VAS, Visual Analogue Scale; PRMDs, playing-related musculoskeletal disorders; PAS Q-DASH, Performing Arts section of the Quick Disabilities of the Arm, Shoulder and Hand Outcome Measure; PDI, Pain Disability Index; PSEQ-2, 2-item short form of the Pain Self-efficacy Questionnaire; NMQ, Nordic Musculoskeletal Questionnaire; IPAQ-SF, International Physical Activity Questionnaire – short form; K-10, Kessler Psychological Distress Scale; HFMPS-SF, Multidimensional Perfectionism Scale – short form; CFQ-11, Chalder Fatigue Scale

The web-based questionnaire includes different research measures (See Additional file 1 and Fig. 2), which have been selected based on a thorough critical review of published research studies and systematic reviews among the performing arts literature, and correspond to possible risk factors associated with the development of PRMDs.

Table 1 describes the primary and secondary outcomes, whereas Table 2 describes the potential risk factors that may be associated with the outcomes.

**Table 1:** Primary and secondary outcomes of the study

Outcome	Case definition (criteria for the health disorder)	Assessment measures	Type of outcome
PRMDs	Any painful musculoskeletal condition that, according to Zaza et al.’s definition (1998), interfere with the ability to play an instrument at the level a participant is accustomed to	Specific question: “Has this painful musculoskeletal condition interfered with your ability to play your instrument at the level to which you are accustomed?”	Primary outcome
MSK conditions	Any condition characterised by pain and limitations in mobility, dexterity and functional ability (according to the World health Organisation)	- NMQ - VAS - PAS Quick DASH - PDI - PSEQ-2	Secondary outcome

*PRMDs* playing-related musculoskeletal disorders, *MSK* musculoskeletal, *NMQ* Nordic Musculoskeletal Questionnaire, *VAS* Visual Analogue Scale, *PAS Quick DASH* Performing Arts section of the Quick Disabilities of the Arm, Shoulder and Hand Outcome Measure, *PDI* Pain Disability Index, *PSEQ-2* 2-item short form of the Pain Self-efficacy Questionnaire

**Table 2:** Measures of the potential risk factors that may be associated with the outcomes

Potential risk factor	Assessment measures
Health status	SRH
Physical activity level	IPAQ-SF
Anxiety and depression	K-10
Perfectionism	HFMPS-SF
Fatigue	CFQ-10

*SRH* Self-Rated Health, *IPAQ-SF* International Physical Activity Questionnaire – short form, *K-10* Kessler Psychological Distress Scale, *HFMPS-SF* Multidimensional Perfectionism Scale – short form, *CFQ-11* Chalder Fatigue Scale

The questionnaire starts with questions about personal background and lifestyle (i.e. age, gender, self-reported weight and height, nationality, smoking status and sleeping habits), as well as practice habits (i.e. number of hours of practice and years of experience), health history (i.e. major past injuries/accident/disorders and current medication) and the Self-Rated Health (SRH) for the assessment of health status [19].

Afterwards, in order to divide the cohorts, the following question is asked: “When did you last experience any painful musculoskeletal condition?” with the following list of possible answers:

1. I currently have a painful musculoskeletal condition (up to one month)
2. 2–3 months ago
3. 4–6 months ago
4. Up to 12 months ago
5. More than 12 months ago
6. I have never had any painful musculoskeletal conditions

Depending on the answer to this question, students are directed to different web pages throughout the questionnaire (See Additional file 1 and Fig. 2):

- Participants with a current painful MSK condition (up to one month) will be asked to answer further questions to describe their current painful MSK condition; the question according to Zaza et al. [8] to identify a PRMD; the Visual Analogue Scale (VAS) to report the intensity of their MSK condition; the Performing Arts Section of the Quick Disabilities of the Arm, Shoulder and Hand Outcome Measure (PAS –Quick DASH) [20] and the Pain Disability Index (PDI) [21–23] to assess their disability; the 2-item short form of the Pain Self-efficacy Questionnaire (PSEQ-2) [24] to assess their self-efficacy; the Nordic Musculoskeletal Questionnaire (NMQ) [25] for the assessment of MSK pain in the last 12 months.
- Participants without any current painful MSK condition but a positive history of it in the last 12 months (2–3 months ago, 4–6 months ago or up to 12 months ago) will be asked to answer the NMQ and the question according to Zaza et al. [8] to identify a PRMD, as well as questions on pain management to investigate how their condition has been treated.
- Participants without any painful MSK condition and participants with a positive history of MSK conditions more than 12 months ago are directly addressed to the next section.

All participants are addressed to the next section, including the following measures:

- International Physical Activity Questionnaire – short form (IPAQ-SF) for the assessment of physical activity participation levels [26].
- Kessler Psychological Distress Scale (K-10) for the assessment of anxiety and stress [27].
- Multidimensional Perfectionism Scale – short form (HFMPS-SF) for the assessment of perfectionism [28–30].
- The Chalder Fatigue Scale (CFQ-11) for the assessment of fatigue [31].

### Data analysis

Descriptive statistics will be used to systematically summarise and present baseline data.

Subgroup analyses based on gender, level of study, hours of practice on reported outcomes will be undertaken using appropriate inferential statistics depending on the type of data and normality checks.

Baseline variables will be categorised to permit the calculation of risk ratios and the 95% confidence intervals (CI) for the development of PRMDs. Tests for trend of relative risk across categories will be carried out using the chi-square test. In order to check for confounders, logistic regression will be performed, adjusting for age, gender and different musical instrument group.

Sample size estimation is reported in Fig. 1.

Both the follow-ups will permit longitudinal change in outcome scores to be generated and compared (longitudinal comparisons) for strength and progression of association, alongside those from absolute outcome scores within several cross-sectional analyses.

For the evaluation of the association amongst the variables (gender, instrument group, age, years of playing, hours of practice, and questionnaires’ scores) and PRMDs, univariate analysis will be performed. Afterwards, all predictor variables that are significantly associated with the occurrence of PRMDs will be included in a multivariate regression model to estimate the mutually adjusted effect of predictors on PRMDs.

## Discussion

To the best of our knowledge, no other longitudinal studies on selected and modifiable risk factors for PRMDs have been available so far; therefore, this study will address the methodological gaps using a longitudinal research design, which could be replicated with other cohorts of students or professionals.

Considering the need for further research, the results of the present study could be used to plan interventions for PRMDs’ prevention based on a risk factor model, with the associated factors being scientifically demonstrated using a longitudinal design.

The results of the present study may help to classify risk factors that can be modified in order to provide an improved conceptual framework for further studies that will more effectively investigate whether reduced injury risk is possible. For instance, while gender, individual physiology, instrument played, and age cannot be modified, lifestyle factors (physical condition, nutrition and health behaviours) and playing behaviours (playing habits, length and intensity of practice time, content and breaks) may be more easily changed [8].

A proper prevention and health awareness could be a potential contribution to a healthier educational context and may reduce the impact of physical and psychological disorders among music students aspiring to become professional musicians 6).

In fact, based on self-reported PRMDs’ rates from the current literature, it seems that the prevalence of PRMDs in music students is relatively unchanged in the last 20–30 years [6] and is still quite high. Injury rates could be related to an insufficient health promotion and injury prevention awareness during music students’ training. This indicates that better results could be obtained by addressing health awareness and attitudes to injury at the university or even at the pre-college.

Therefore, new strategies that can provide useful instruction on the care of the body and injury prevention may be developed, taking into consideration the findings of the present study and the latest research findings from performing arts medicine into becoming effective and functional resources.

The study findings and its potential for translation into practice will be disseminated in various forms including but not limited to, presentations at international conferences, peer-reviewed journal publications, newsletters and social media avenues, and by means of communication and marketing departments of participating schools and universities.

Dissemination of findings amongst schools of music and academies is one of the investigation’s key targets. In order to ensure that it is successfully achieved, we have planned to produce and deliver summaries of the findings to all participant centres, as well as to circulate flyers via email and social media in order to reach all students who have participated in the research study and all target students who may be interested.

Furthermore, a dissemination strategy, including dialogue with stakeholders from civic organisations, university schools of music and academies, labour organisations, educational institutions and policy makers in different countries has also been planned.

### Limitations

Despite the manifold benefits associated with this investigation, there are limitations to be aware of when considering the study design.

Firstly, this study will use online-based administration of questionnaires that has the benefit of being able to reach a larger population sample in a time- and cost-efficient way, but this could also represent a high risk of data attrition; students are usually considered to be internet users but nowadays they are constantly burdened by messages and emails that can easily be deleted or ignored.

Secondly, self-reported data might be limited by the fact that results cannot be independently and objectively verified. In fact, self-report methods can contain potential bias (i.e. remembering or not remembering experiences or events that occurred in the past; attributing positive or negative outcomes to events due to external forces; over- or underestimating events or experiences).

Thirdly, the data will be obtained through a study design of 6-monthly self-reported questionnaires, which is inevitably subject to recall bias, but on the other hand is the cost-effective way to collect data from large numbers of people. The limitations but also advantages of self-reported data will be considered and necessary levels of caution will be applied when interpreting the results.

It could be argued that participants should be observed and tested with higher frequency to more accurately investigate the time frame of developing PRMDs and associated symptoms, but this would not be feasible for logistical purposes. We think that the proposed time interval between assessments is appropriate in order to pick out important and more permanent PRMDs and not transient ones that may come and go as part of normal daily activities.

Moreover, although participants may forget to report the onset of PRMDs dating several months back when only required to do so every six months, sometimes PRMDs may be chronic and recurrent. We believe that any bias is small and only those participants who experience transient pain or the least MSK conditions will be missed.

In addition, we believe that the response rate may be negatively influenced by more frequent assessment intervals, particularly when participants are reached via email as is required in this study.

Finally, there are also different recall periods among the questionnaires during the follow-ups. More specifically, the Quick Dash and IPAQ refer to the previous 7 days, CFQ-11, as well as K10 refer to the last 30 days and finally in the HFMPS-SF, there is no specific time reference.

Nonetheless, this study may represent an effort to improve upon previous methods of investigation (i.e. cross-sectional study designs) associated with factors for increased risk of PRMDs among musicians, and a step forward in education and employment within musical contexts.

## Additional file

Additional file 1: RISMUS questionnaire.pdf. The web-based questionnaire of the longitudinal study. (PDF 1050 kb)

## Abbreviations

CFQ-11: Chalder Fatigue Scale
HFMPS-SF: Multidimensional Perfectionism Scale – short form
IPAQ-SF: Physical Activity Questionnaire – short form
K-10: Kessler Psychological Distress Scale
MSK: Musculoskeletal
NMQ: Nordic Musculoskeletal Questionnaire
PAS –Quick DASH: Performing Arts Section of the Quick Disabilities of the Arm, Shoulder and Hand Outcome Measure
PDI: Pain Disability Index
PRMDs: Playing-related musculoskeletal disorders
PSEQ-2: 2-item short form of the Pain Self-efficacy Questionnaire
SRH: Self-Rated Health
VAS: Visual Analogue Scale
