# Supplementary material for: A longitudinal investigation of the factors associated with increased RISk of playing-related musculoskeletal disorders in MUsic students (RISMUS): a study protocol
Source: BMC Musculoskelet Disord. 2019 Feb 8;20:64. doi: 10.1186/s12891-019-2440-4 (PMC6368778; doi:10.1186/s12891-019-2440-4)
Supplement: Supplementary file 1 — RISMUS questionnaire.pdf. The web-based questionnaire of the longitudinal study. (PDF 1050 kb) [file 12891_2019_2440_MOESM1_ESM.pdf]

RISMUS _RISK AMONG MUSIC STUDENTS_

Background information

* 1. To ensure anonymity, please create your own code according to the following information:
- Day of your birthday
- First three letters of your favourite composer

Example: if your birthday is 3rd January, 1990 and your favourite composer is Beethoven, your code would be 03bee

* 2. What is your gender?

- Female
- Male
- Other

* 3. How old are you?

4. Height and weight

How tall are you? (in cm)

How much do you weigh? (in kg)

5. What is your nationality?

* 6. What is your main instrument?

Other (please specify)

* 7. What is your academic level?

- Pre-College Conservatory and Junior Program
- Undergraduate (Bachelor's degree) - Year 1, 2
- Undergraduate (Bachelor's degree) - Year 3 and 4
- Postgraduate (Master's degree) - Year 1 and 2
- Postgraduate (Master's degree) - Year 3 and 4
- Gap year experience Programme
- Other

Other (please specify)

8. Musical background

How long have you been playing your instrument?
Years:

How many hours do you spend practising every day on average?
Hours per day:

9. During the last 6 months,

	Yes	No
Have you changed your teacher or Professor?	◯	◯
Have you change your instrument?	◯	◯
Have you changed your technique/intrument set-up?	◯	◯

10. Please rate your perceived exertion after playing your instrument 45 minutes without rest (how tired you feel in general):

Very low Very high

11. Before practising or performing, do you do any preparatory exercises (stretching and/or breathing WITHOUT your instrument)?

◯ No

◯ Yes
How much time in minutes do you spend doing preparatory exercises on average?
Minutes:

12. Do you take active breaks to restore body posture or to allow active muscles to relax?

◯ Yes

◯ No

13. If yes,

How often do you take them?

How long do they last?
Minutes:

14. In general, would you say your health is:
(according to World Health Organisation, the definition of health is: "A state of complete physical, mental and social well-being and not merely the absence of disease or infermity")

◯ Excellent

◯ Very good

◯ Good

◯ Fair

◯ Poor

15. On average, how many hours of sleep do you get a night?

Hours per night (on average):

16. Do you smoke?

- ◯ Yes
- ◯ No

17. Smoking habits

On average, how many cigarettes do you smoke per day?

[ ]

How long have you been smoking?
Years/Months:
(Please specify years or months)

[ ]

* 18. Do you currently take any medication?

- ◯ No
- ◯ Yes

Please specify:

[ ]

* 19. During the last 12 months, have you had any injury and/or an accident resulted into hospitalisation and surgery that prevented you to do your normal activities?

- ◯ No
- ◯ Yes

Please specify:

[ ]

* 20. During the last 12 months, have you had any surgery of the upper limbs and/or the spine?

- ◯ No
- ◯ Yes

* 21. During the last 12 months, have you had any diagnosed psychological disorder or emotional distress?

- ◯ No
- ◯ Yes

Please specify:

[ ]

* 22. During the last 12 months, have you had any diagnosed neurological or rheumatic disorder?

- ◯ No
- ◯ Yes

Please specify:

[ ]

Musculoskeletal disorders

* 1. When did you LAST experience a painful musculoskeletal condition?

- ◯ I currently have a painful musculoskeletal condition (up to one month)
- ◯ 2-3 months ago
- ◯ 4-6 months ago
- ◯ Up to 12 months ago
- ◯ More than 12 months ago
- ◯ I have never had any painful musculoskeletal conditions

RISMUS _RISK AMONG MUSIC STUDENTS_

COHORT 2 <1mo [type]

According to your answer, it seems like you currently have a painful musculoskeletal condition. Please focus on this problem when answering the following questions.

1. How long have you had your current painful musculoskeletal condition?

- ◯ 0-1 week
- ◯ 1-2 weeks
- ◯ 3-4 weeks
- ◯ 4-5 weeks
- ◯ 6-8 weeks
- ◯ 9-11 weeks
- ◯ 3-6 months
- ◯ 6-9 months
- ◯ 9-12 months
- ◯ over 1 year

2. Which word or words would you use to describe the pattern of your painful musculoskeletal condition?

- ◯ continuous steady constant
- ◯ rhythmic periodic intermittent
- ◯ brief momentary transient

3. Has this painful musculoskeletal condition interfered with your ability to play your instrument at the level to which you are accustomed?
In other words: has this painful musculoskeletal condition had a negative impact on your way of playing as well as you would like if you did not have it?

- ◯ Yes
- ◯ No

4. Do you think this painful musculoskeletal condition was caused by your playing?

- ◯ Yes
- ◯ No
- ◯ I don't know

5. In your opinion, what are the main causes of your painful musculoskeletal condition?

[ ]

6. Please rate the severity of your painful musculoskeletal condition:

0 No pain — 10 Worst possible pain [ ]

7. Please indicate the type of your painful musculoskeletal condition:

- ☐ Aching
- ☐ Annoying
- ☐ Burning
- ☐ Cramping
- ☐ Numbness
- ☐ Stiffness
- ☐ Soreness
- ☐ Stabbing
- ☐ Swelling
- ☐ Tingling
- ☐ Weakness
- ☐ I don't know
- ☐ Other (please specify)

[ ]

In these areas, you can see the approximate position of the different anatomical parts of the body. Limits are not sharply defined, and certain parts overlap.

Click on the part(s) in which you have or have had your painful musculoskeletal condition during the last month.
<u>You can choose more than one part</u>.

8. UPPER LIMB

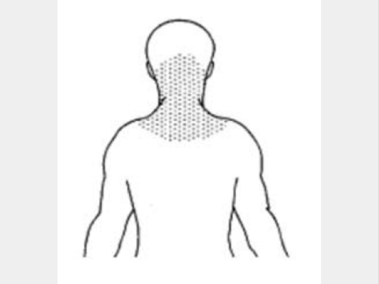
NECK

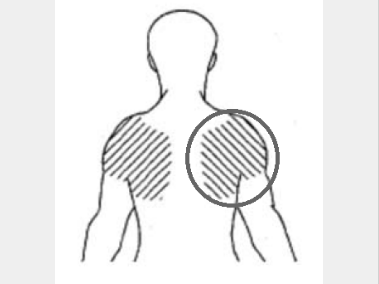
RIGHT SHOULDER

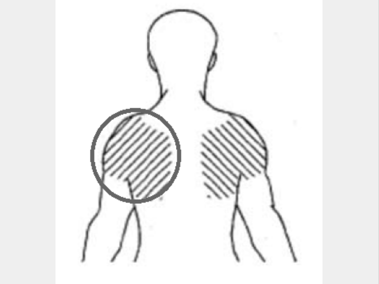
LEFT SHOULDER

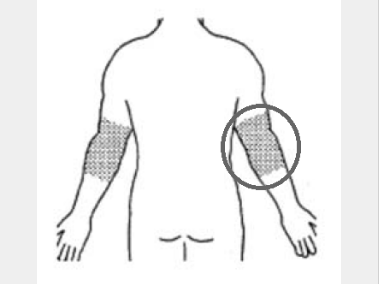
RIGHT ELBOW

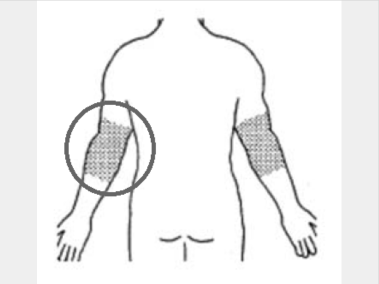
LEFT ELBOW

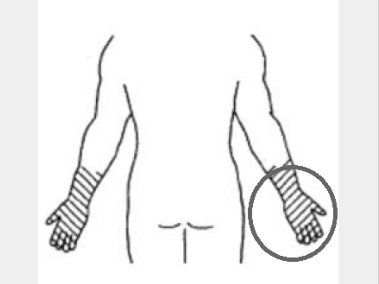
RIGHT WRIST/HAND

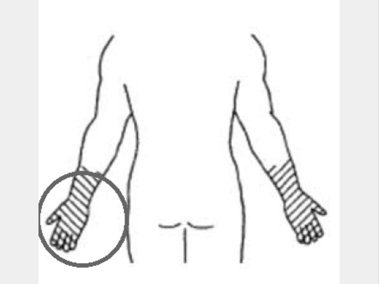
LEFT WRIST/HAND

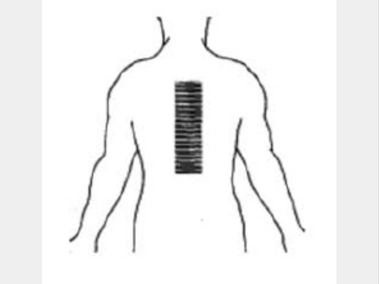
UPPER BACK

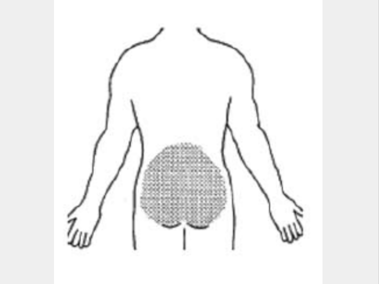
LOW BACK

9. LOWER LIMB

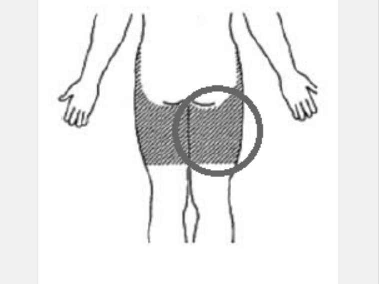
RIGHT HIP/THIGH

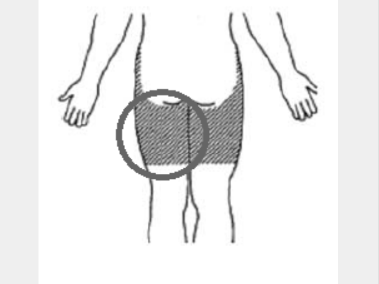
LEFT HIP/THIGH

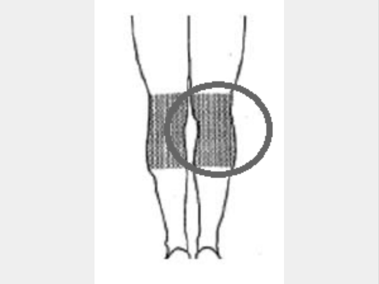
RIGHT KNEE

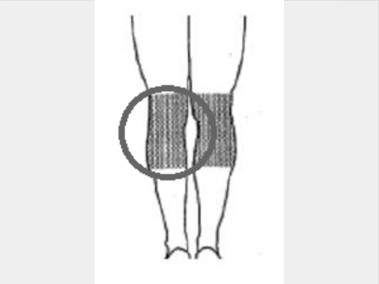
LEFT KNEE

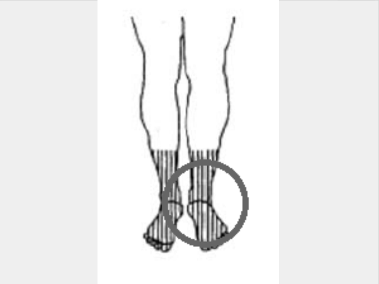
RIGHT ANKLE/FOOT

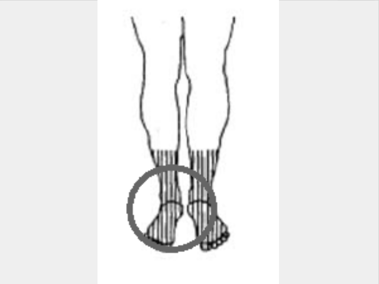
LEFT ANKLE/FOOT

PERFORMING ARTS

The following questions relate to the impact of your arm, shoulder or hand problem on playing your musical instrument.

Please choose the number that best describes your physical ability in the past week.

1. During the last week, did you have any difficulty:

	1 No difficulty	2 Mild difficulty	3 Moderate difficulty	4 Severe difficulty	5 Unable
Playing your instrument in your usual way?	○	○	○	○	○
Playing your musical instrument because of arm, shoulder or hand pain?	○	○	○	○	○
Playing your instrument as well as you would like?	○	○	○	○	○
Spending your usual amount of time practising or playing your instrument?	○	○	○	○	○

COHORT 2 <1mo [PDI]

The rating scales below are designed to measure the degree to which aspects of your life are disrupted by pain. In other words, we would like to know how much pain is preventing you from doing what you would normally do or from doing it as well as you normally would.

Respond to each category indicating the overall impact of pain in your life, not just when pain is at its worst.
For each of the 7 categories of life activity listed, please rate the level of disability you typically experience. A score of 0 means no disability at all, and a score of 10 signifies that all of the activities in which you would normally be involved have been totally disrupted or prevented by your pain.

1. FAMILY/HOME RESPONSABILITIES
This category refers to activities of the home or family. It includes chores or duties performed around the house (e.g. yard work) and errands or favors for other family members

0 No disability — 10 Worst Disability

2. RECREATION
This disability includes hobbies, sports, and other similar leisure time activities

0 No disability — 10 Worst Disability

3. SOCIAL ACTIVITY
This category refers to activities, which involve participation with friends and acquaintances other than family members. It includes parties, theater, concerts, dining out, and other social functions

0 No disability — 10 Worst Disability

4. OCCUPATION
This category refers to activities that are part of or directly related to one’s job. This includes non-paying jobs as well, such as that of a homemaker or volunteer

0 No disability — 10 Worst Disability

5. SEXUAL BEHAVIOUR
This category refers to the frequency and quality of one’s sex life

0 No disability — 10 Worst Disability

6. SELF-CARE
This category includes activities, which involve personal maintenance and independent daily living (e.g. taking a shower, driving, getting dressed, etc.)

0 No disability — 10 Worst Disability

7. LIFE SUPPORT ACTIVITY
This category refers to basic life supporting behaviours such as eating, sleeping and breathing

0 No disability — 10 Worst Disability

Now please rate how confident you are that you can do the following things at present, despite the pain.
Remember, this questionnaire is not asking whether or not you have been doing these things, but rather how confident you are that you can do them at present, despite the pain.

8. I can do some form of work, despite the pain (‘‘work’’ includes housework and paid and unpaid work):

0 Not at all confident — 6 Completely confident

9. I can live a normal lifestyle, despite the pain:

0 Not at all confident — 6 Completely confident

RISMUS _RISK AMONG MUSIC STUDENTS_

COHORT 2 <12mo [Nordic]

Now you should try to remember any trouble (such as ache, pain, discomfort, numbness) you have had DURING THE LAST 12 MONTHS.

In the picture below you can see the approximate position of the parts of the body referred to in the questions.
Limits are not sharply defined, and certain parts overlap. You should decide for yourself in which part you have or have had your trouble (ache, pain, discomfort).You may be in doubt as to how to answer, but please do your best anyway.

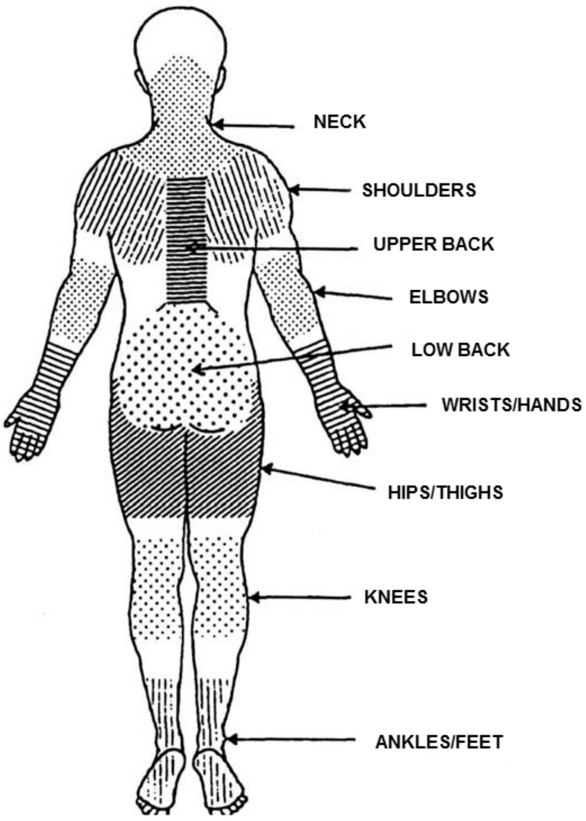

1. Please answer by choosing the appropriate box-one cross for ALL PARTS in which you have or have had any trouble (ache, pain, discomfort).

	Neck	Shoulders	Elbows	Wrists/hands	Upper back	Low back	Hips/thighs	Knees	Ankles/Feet
Have you at any time during the last 12 months had trouble (ache, pain, discomfort) in:	☐	☐	☐	☐	☐	☐	☐	☐	☐
Have you at any time during the last 12 months been prevented from doing your normal work (at home or away from home) because of the trouble in:	☐	☐	☐	☐	☐	☐	☐	☐	☐
Have you at any time during the last 12 months been seen by a doctor, physiotherapist, chiropractor or other such person because of trouble in:	☐	☐	☐	☐	☐	☐	☐	☐	☐

2. Have you at any time during the last 7 days had trouble (ache, pain, discomfort) in:

☐ Neck	☐ Wrists/Hands	☐ Hips/Thighs
☐ Shoulders	☐ Upper back	☐ Knees
☐ Elbows	☐ Low back	☐ Ankles/Feet

COHORT 2 <12mo

1. According to your answers, it seems like you do not currently have any painful musculoskeletal condition.
Could you please indicate what you have done to manage it?

2. Did this painful musculoskeletal condition interfer with your ability to play your instrument at the level to which you are accustomed?

In other words: did this painful musculoskeletal condition have a negative impact on your way of playing as well as you would like if you did not have it?

- ◯ Yes
- ◯ No

3. Do you think this painful musculoskeletal condition was caused by your playing?

- ◯ Yes
- ◯ No
- ◯ I don't know

4. In your opinion, what were the main causes of your painful musculoskeletal condition?

COHORT 1 >12mo

1. How many years have passed since you have not had any painful musculoskeletal condition?

Years:

[ ]

Physical activity

We are interested now in finding out about the kinds of physical activities that people do as part of their everyday lives.
The questions will ask you about the time you spent being physically active in the last 7 days. Please answer each question even if you do not consider yourself to be an active person. Please think about the activities you do at work, as part of your house and yard work, to get from place to place, and in your spare time for recreation, exercise or sport.
If you are normally active but in the previous 7 days you did not do any physical activity, please think about the most recent typical week.

1. Think about all the VIGOROUS activities that you did in the last 7 days.
Vigorous physical activities refer to activities that take hard physical effort and make you breathe much harder than normal. Think only about those physical activities that you did for at least 10 minutes at a time.

During the last 7 days, on how many days did you do VIGOROUS physical activities like heavy lifting, digging, aerobics, or fast bicycling?

○ No vigorous physical activities (skip to question 3)

○ Days per week: [ ]

2. How much time did you usually spend doing vigorous physical activities on one of those days?

Minutes per day: [ ]

3. Think about all the MODERATE activities that you did in the last 7 days. Moderate activities refer to activities that take moderate physical effort and make you breathe somewhat harder than normal. Think only about those physical activities that you did for at least 10 minutes at a time.

During the last 7 days, on how many days did you do MODERATE physical activities like carrying light loads, bicycling at a regular pace, or doubles tennis?

Do not include walking.

○ No moderate physical activities (skip to question 5)

○ Days per week: [ ]

4. How much time did you usually spend doing MODERATE physical activities on one of those days?

Minutes per day: [ ]

5. Think about the time you spent WALKING in the last 7 days. This includes at work and at home, walking to travel from place to place, and any other walking that you have done solely for recreation, sport, exercise, or leisure.
During the last 7 days, on how many days did you walk for at least 10 minute at a time?

○ No walking (skip to question 7)

○ Days per week: [ ]

6. How much time did you usually spend WALKING on one of those days?

Minutes per day:

7. The last question is about the time you spent SITTING on week days during the last 7 days or in the most recent typical week.

Include time spent at work, at home, while doing course work and during leisure time. This may include time spent sitting at a desk, visiting friends, reading, or sitting or lying down to watch television.

During the last 7 days, how much time did you spend SITTING on a week day?

Hours per day:

Your feelings

This section will ask you how you have been feeling over the last 30 days.
Please choose the answer that best represents how you have been.

1. During the last 30 days, about how often did you feel:

	None of the time	A little of the time	Some of the time	Most of the time	All of the time
tired out for no good reason?	◯	◯	◯	◯	◯
nervous?	◯	◯	◯	◯	◯
so nervous that nothing could calm you down?	◯	◯	◯	◯	◯
hopeless?	◯	◯	◯	◯	◯
restless or fidgety?	◯	◯	◯	◯	◯
so restless you could not sit still?	◯	◯	◯	◯	◯
depressed?	◯	◯	◯	◯	◯
that everything was an effort?	◯	◯	◯	◯	◯
so sad that nothing could cheer you up?	◯	◯	◯	◯	◯
worthless?	◯	◯	◯	◯	◯

Your personal characteristics and traits

Listed below are a number of statements concerning personal characteristics and traits.

1. Read each item and decide whether you agree or disagree and to what extent.

	1 Disagree	2	3	4	5	6	7 Agree
One of my goals is to be perfect in everything I do	◯	◯	◯	◯	◯	◯	◯
Everything that others do must be of top-notch quality	◯	◯	◯	◯	◯	◯	◯
The better I do, the better I am expected to do	◯	◯	◯	◯	◯	◯	◯
I strive to be as perfect as I can be	◯	◯	◯	◯	◯	◯	◯
It is very important that I am perfect in everything I attempt	◯	◯	◯	◯	◯	◯	◯
I have high expectations for the people who are important to me	◯	◯	◯	◯	◯	◯	◯
I demand nothing less than perfection of myself	◯	◯	◯	◯	◯	◯	◯
I can’t be bothered with people who won’t strive to better themselves	◯	◯	◯	◯	◯	◯	◯
Success means that I must work even harder to please others	◯	◯	◯	◯	◯	◯	◯
If I ask someone to do something, I expect it to be done flawlessly	◯	◯	◯	◯	◯	◯	◯
I cannot stand to see people close to me make mistakes	◯	◯	◯	◯	◯	◯	◯
I must work to my full potential at all times	◯	◯	◯	◯	◯	◯	◯
My family expects me to be perfect	◯	◯	◯	◯	◯	◯	◯
People expect nothing less than perfection from me	◯	◯	◯	◯	◯	◯	◯
People expect more from me than I am capable of giving	◯	◯	◯	◯	◯	◯	◯

RISMUS _RISK AMONG MUSIC STUDENTS_

Your tiredness

We would like to know more about any problems you have had with feeling tired, weak or lacking in energy in the last 30 days.

1. Please answer all the questions by choosing the answer which applies to you most closely.
If you have been feeling tired for a long while, then compare yourself to how you felt when you were last well.

	Less than usual	No more than usual	More than usual	Much more than usual
Do you have problems with tiredness?	○	○	○	○
Do you need to rest more?	○	○	○	○
Do you feel sleepy or drowsy?	○	○	○	○
Do you have problems starting things?	○	○	○	○
Do you lack energy?	○	○	○	○
Do you have less strength in your muscles?	○	○	○	○
Do you feel weak?	○	○	○	○
Do you have difficulties concentrating?	○	○	○	○
Do you make slips of the tongue when speaking?	○	○	○	○
Do you find it more difficult to find the right word?	○	○	○	○

2. How is your memory?

- ○ Better than usual
- ○ No worse than usual
- ○ Worse than usual
- ○ Much worse than usual

End of the questionnaire

THIS IS THE END OF THE QUESTIONNAIRE!

Many thanks for your responses.

PLEASE REMEMBER THAT YOU WILL BE CONTACTED AGAIN FOR A FOLLOW-UP IN 6 MONTHS!

If you have questions about this project or if you have a research related problem, you may contact the researcher, Cinzia Cruder at Ccruder@qmu.ac.uk

As researcher, I am not qualified to provide counselling services or medical advice. If you feel upset after completing the questionnaire, or find that some questions or aspects of the study triggered distress, talking with a qualified clinician may help.
If you feel you would like health assistance in relation to your musical practice, please visit BAPAM's website: www.bapam.org.uk

The British Association for Performing Arts Medicine (BAPAM) is a healthcare charity giving medical advice to people working and studying in the performing arts. BAPAM help you overcome (and preferably avoid) playing-related health problems, and are dedicated to sharing knowledge about healthy practice.
